# *TERT* promoter Mutation and Its Association with Clinicopathological Features and Prognosis of Papillary Thyroid Cancer: A Meta-analysis

**DOI:** 10.1038/srep36990

**Published:** 2016-11-11

**Authors:** Chunping Liu, Zeming Liu, Tianwen Chen, Wen Zeng, Yawen Guo, Tao Huang

**Affiliations:** 1 Department of Breast and Thyroid Surgery, Union Hospital, Tongji Medical College, Huazhong University of Science and Technology, Wuhan, People’s Republic of China; 2 Department of Ophthalmology, Zhongnan Hospital, Wuhan University, Wuhan, Hubei, China

## Abstract

We performed a meta-analysis to elucidate the associations of the clinicopathological characteristics and prognostic factors of papillary thyroid cancer (PTC) with *TERT* promoter mutations. A literature search was performed of the PubMed and EMBASE databases using Medical Subject Headings and keywords. Individual study-specific odds ratios (ORs) and confidence intervals (CIs) were calculated. The average prevalence rate of *TERT* promoter mutations was 10.1%. TERT promoter mutations occurred more frequently in patients with larger tumors (p = 0.003). *TERT* promoter mutations were associated with advanced stage (OR = 3.11, 95% CI = 2.22–4.36), lymph node metastasis (OR = 1.82, 95% CI = 1.12–2.96), distant metastasis (OR = 4.18, 95% CI = 1.61–10.81), *BRAF* mutation positivity (OR = 2.71, 95% CI = 1.45–3.24), recurrence (OR = 3.91, 95% CI = 1.83–8.34), and mortality (OR = 8.13, 95% CI = 3.77–17.53). The associations of *TERT* promoter mutations with extrathyroidal invasion (OR = 1.98, 95% CI = 0.96–4.07), unifocality (OR = 1.36, 95% CI = 0.90–2.07), and vascular invasion (OR = 1.45, 95% CI = 0.92–2.30) were not significant. *TERT* promoter mutations are closely associated with aggressive clinicopathological characteristics and poorer prognosis in PTC.

Thyroid cancer is the most common malignant tumor of the endocrine system, and its global incidence has rapidly increased in recent decades^1^. The most common thyroid malignancy is papillary thyroid cancer (PTC), which is derived from the follicular epithelium^2^. Although conventional surgery with adjuvant radioiodine (^131^I) therapy has been the main treatment for PTC, it is often not curative. Recently, improved understanding of the molecular pathogenesis of PTC has increased the prospects for developing more effective therapies for this cancer.

Telomerase is responsible for the elongation of telomeric DNA. However, it can also lead to the infinite proliferation of malignant cells by stabilizing telomere length^3^,^4^. Telomerase reverse transcriptase (TERT) is considered a predominant determinant for controlling the activity of telomerase. Derepression of *TERT* transcription is known to induce telomerase activation and consequent malignant transformation^5^,^6^. In recent years, the mechanism underlying *TERT* transcriptional activation in cancer cells has been an active area of investigation.

More recently, a few studies have demonstrated the presence of *TERT* promoter mutations in PTC^6^,^7^. However, the diagnostic and prognostic utility of *TERT* promoter mutations in PTC remains controversial and requires additional investigation^7^,^8^. Therefore, we performed a systematic review and meta-analysis to elucidate the association of *TERT* promoter mutations with clinicopathological characteristics and prognosis in PTC.

## Results

### Literature Searches and Study Characteristics

**Figure 1:**
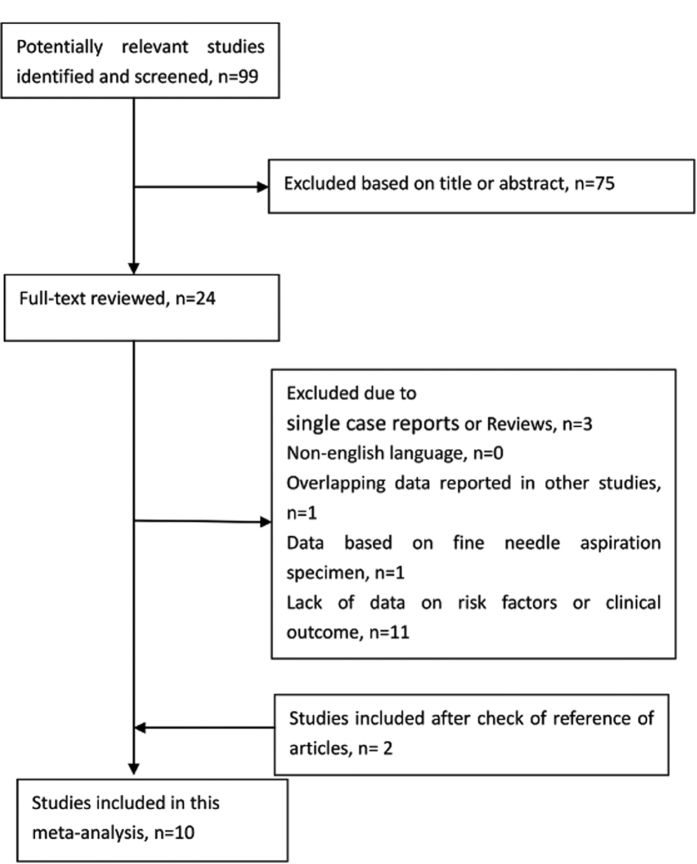
The study selection process.

Figure 1 shows the study selection process. A total of 99 abstracts and titles were obtained through electronic searches. Of these abstracts and papers, 24 full-text papers were deemed relevant and examined in detail. Following this detailed review, 10 studies met our inclusion/exclusion criteria^6^,^7^,^8^,^9^,^10^,^11^,^12^,^13^,^14^,^15^, and these studies contributed 2537 patients with PTC to the meta-analysis. The main features of the 10 eligible studies that investigated prognostic factors are summarized in Table 1. Among these studies, six studies evaluated extrathyroidal invasion, and three studies evaluated tumor size. Data regarding TNM stage, multifocality, vascular invasion, lymph node metastasis, distant metastasis, and *BRAF* V600E mutations were reported in seven, four, four, nine, four, and five studies, respectively (Tables 1 and 2). The funnel plots for each outcome did not suggest the presence of publication bias (data not shown).

### Meta-analysis of the Effects of *TERT* promoter mutations on Clinicopathological Features

**Figure 2:**
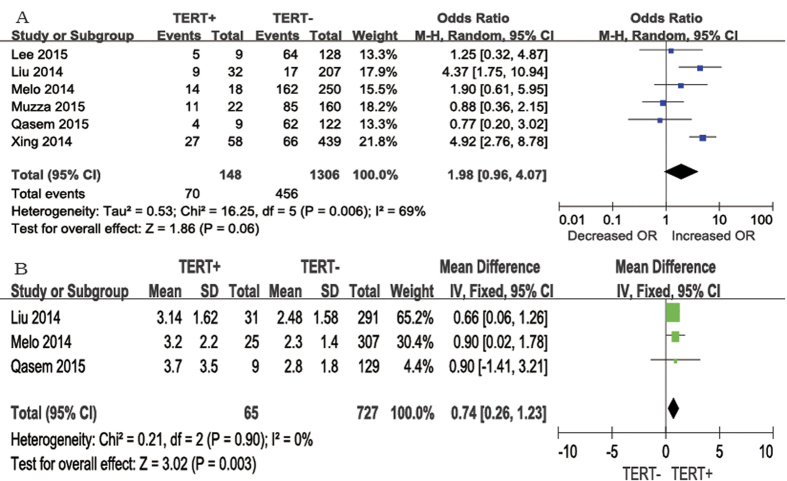
The odds ratios (ORs), Mean Difference (MD) with 95% confidence intervals (CIs) for the association between TERT mutation and extrathyroidal invasion (**A**) and larger tumor size (cm) (**B**) respectively in patients with PTC. M-H, Mantel-Haenszel; IV, inverse variance.

In each category of prognostic factors, the pooled ORs were higher in patients with *TERT* promoter mutations than in those with the wild-type gene. Six studies presented clinical data including extrathyroidal invasion. Extrathyroidal invasion was present in 70 (47.3%) of 148 patients with *TERT* promoter mutations and in 456 (34.9%) of 1306 patients without *TERT* promoter mutations (Fig. 2A). The average odds ratio (OR) from the six studies was 1.98 (95% confidence interval [CI] = 0.96–4.07). The heterogeneity of the data was significant (P = 0.006), and the *I*^2^ estimate of the variance between the studies was 69%. According to our analysis, the difference between the occurrence of extrathyroidal invasion according to *TERT* promoter mutation positivity was not significant (P = 0.06).

We analyzed three studies that reported tumor size (Fig. 2B). A fixed-effects model was adopted, as heterogeneity was not significant between tumor size and *TERT* promoter mutations (P = 0.90, *I*^2^ = 0%). According to our analysis, tumor size was larger in patients with *TERT* promoter mutations than in those without *TERT* promoter mutations. The mean difference was 0.74 cm (95% CI = 0.26–1.23, P = 0.003).

**Figure 3:**
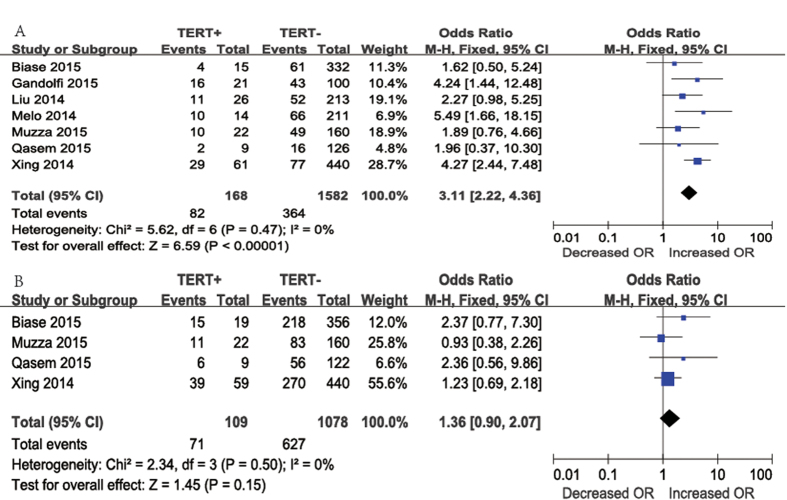
The odds ratios (ORs) with 95% confidence intervals (CIs) for the association between TERT mutation and advanced TNM stage(III/IV) (**A**) and unifocality (**B**) in patients with PTC. M-H, Mantel-Haenszel.

TNM stage was reported for patients in seven studies (Fig. 3A). Advanced TNM stage (III/IV) was identified in 82 (48.8%) of 168 patients with *TERT* promoter mutations and in 364 (23.0%) of 1582 patients without *TERT* promoter mutation*s*. A fixed-effects model was adopted because heterogeneity was not significant between TNM stage and *TERT* promoter mutations (P = 0.47, *I*^2^ = 0%). According to our analysis, advanced TNM stage (III/IV) occurred more frequently in patients with *TERT* promoter mutations than in those without *TERT* promoter mutations. The overall OR was 3.11 (95% CI = 2.22–4.36, P < 0.00001).

Regarding patients with unifocality (Fig. 3B), four studies were included in the meta-analysis. Unifocality was present in 71 (65.1%) of 109 patients with *TERT* promoter mutations and in 627 (58.2%) of 1078 patients without *TERT* promoter mutations. The overall OR was 1.36 (95% CI = 0.90–2.07). A fixed-effects model was adopted, as heterogeneity was not significant between multifocality and *TERT* promoter mutations (P = 0.50, *I*^2^ = 0%). According to our results, there was no significant association between *TERT* promoter mutations and tumor foci (P = 0.15).

**Figure 4:**
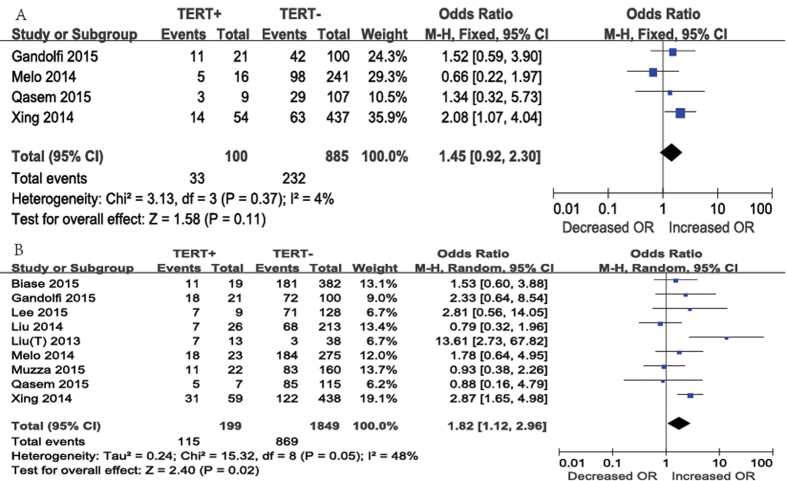
The odds ratios (ORs) with 95% confidence intervals (CIs) for the association between TERT mutation and vascular invasion (**A**) and lymph node metastasis (**B**) in patients with PTC. M-H, Mantel-Haenszel.

Vascular invasion was reported in four studies (Fig. 4A). Vascular invasion was present in 33 (33%) of 100 patients with *TERT* promoter mutations and in 232 (26.2%) of 885 patients without *TERT* promoter mutations. A fixed-effects model was adopted, as heterogeneity was not significant between vascular invasion and *TERT* promoter mutations (P = 0.37, *I*^2^ = 4%). According to our results, the difference in the occurrence of vascular invasion according to *TERT* promoter mutation positivity was not significant. The overall OR was 1.45 (95% CI = 0.92–2.30, P = 0.11).

Regarding cases of lymph node metastasis (Fig. 4B), nine studies were included in the meta-analysis. Lymph node metastasis was present in 115 (57.8%) of 199 patients with *TERT* promoter mutations and in 869 (47.0%) of 1849 patients without *TERT* promoter mutations. The overall OR was 1.82 (95% CI, 1.12–2.96). A random-effects model was adopted because the heterogeneity of the data was significant (P = 0.05), and the *I*^2^ estimate of the variance between the studies was 48%. According to our analysis, lymph node metastasis occurred more frequently in patients with *TERT* promoter mutations than in those without *TERT* promoter mutations (P = 0.02).

**Figure 5:**
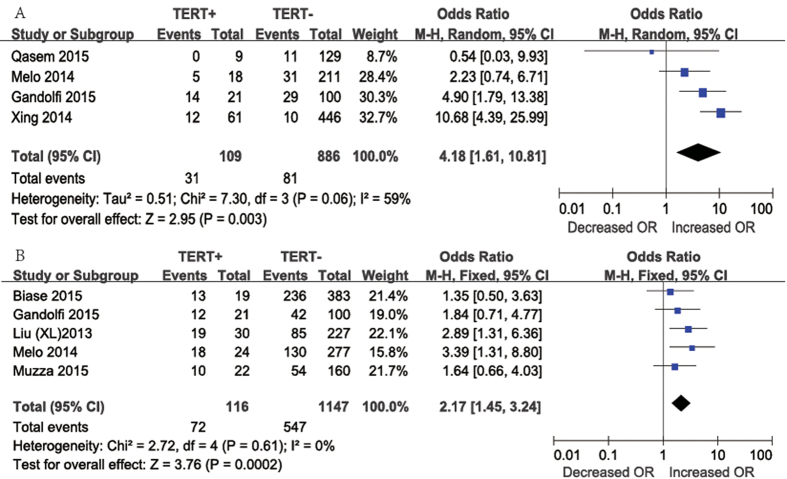
The odds ratios (ORs) with 95% confidence intervals (CIs) for the association between TERT mutation and distant metastasis (**A**) and positive *BRAF* mutation (**B**) in patients with PTC. M-H, Mantel-Haenszel.

Concerning the presence of distant metastasis (Fig. 5A), four studies were included in the meta-analysis. Distant metastasis was present in 31 (28.4%) of 109 patients with *TERT* promoter mutations and in 81 (9.1%) of 886 patients without *TERT* promoter mutations. The overall OR was 4.18 (95% CI, 1.61–10.81). A random-effects model was adopted because the heterogeneity of the data was significant (P = 0.06), and the *I*^2^ estimate of the variance between the studies was 59%. According to our analysis, distant metastasis occurred more frequently in patients with *TERT* promoter mutations than in those without *TERT* promoter mutations (P = 0.003).

Regarding patients with *BRAF* mutations (Fig. 5B), five studies were included in our meta-analysis. *BRAF* mutation positivity was present in 72 (62.1%) of 116 patients with *TERT* promoter mutations and in 547 (47.7%) of 1147 patients without *TERT* promoter mutations. The overall OR was 2.17 (95% CI = 1.45–3.24). A fixed-effects model was adopted because the heterogeneity of the data was not significant (P = 0.61), and the *I*^2^ estimate of the variance between the studies was 0%. According to our analysis, *BRAF* mutations occurred more frequently in patients with *TERT* promoter mutations than in those without *TERT* promoter mutations (P = 0.0002).

### Meta-analysis of the Effect of *TERT* Mutation on Persistent/Recurrence and Mortality

**Figure 6:**
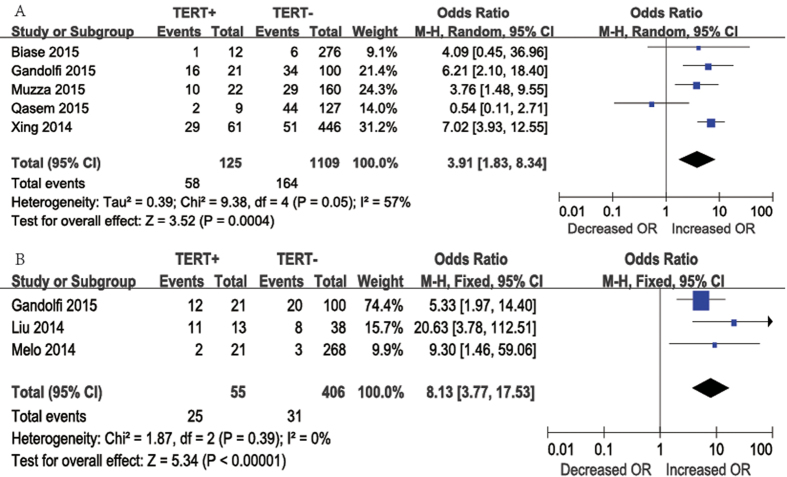
The odds ratios (ORs) with 95% confidence intervals (CIs) for the association between TERT mutation and persistence/recurrence (**A**) and mortality (**B**) in patients with PTC. M-H, Mantel-Haenszel.

Concerning persistence/recurrence, five studies were included in this meta-analysis. Persistence/recurrence occurred in 58 (46.4%) of 125 patients with *TERT* promoter mutations and in 164 (14.8%) of 1109 patients without *TERT* promoter mutations. A random-effects model was adopted because the heterogeneity of the data was significant (P = 0.05), and the *I*^2^ estimate of the variance between the studies was 57%. According to our analysis, persistent/recurrence was more frequent in patients with *TERT* promoter mutations than in those without mutations. The overall OR was 3.91 (95% CI = 1.83–8.34), and the P-value was 0.0004 (Fig. 6A). Furthermore, individual studies were assessed further by sequentially excluding each study from our meta-analysis. In this manner, we found that *I*^2^ was 0% when we excluded the study by Qasem, but this did not occur when other articles were excluded; thus, we concluded that the heterogeneity was mainly caused by this particular study.

Mortality data were reported in three studies (Fig. 6B). Mortality occurred in 25 (45.5%) of 55 patients with *TERT* promoter mutations and in 31 (7.6%) of 406 patients without *TERT* promoter mutations. A fixed-effects model was adopted, as heterogeneity was not significant between mortality and *TERT* promoter mutations (P = 0.39, *I*^2^ = 0%). According to our analysis, mortality was more frequent among patients with *TERT* promoter mutations than among those without mutations. The overall OR was 8.13 (95% CI = 3.77–17.53, P < 0.00001).

### Subgroup Analyses of the Effects of *TERT* promoter mutations on Aggressive Clinicopathological Features and Prognostic Factors

Subgroup analysis was conducted according to the patients’ country of origin to investigate potential sources of heterogeneity and to assess whether the effects of *TERT* promoter mutation*s* on aggressive clinicopathological features and poor prognosis of PTC were associated with geographic regions (Table 3). The effect estimates were broadly consistent among the subgroups that were analyzed. Heterogeneity was markedly decreased in the subgroup analyses of extrathyroidal invasion, distant metastasis, and recurrence. However, this may have been caused by two Asian countries (Saudi Arabia and Korea) being distantly located geographically and being different in many aspects. The two studies involved were among the smallest in our meta-analysis, and the total number of cases and relatively low prevalence of *TERT* promoter mutations led to a lack of statistical power. The results of the subgroup analyses also indicated that *TERT* promoter mutation*s* were not significantly associated with some high-risk tumor features (e.g., extrathyroidal extension and lymph node metastasis) of PTC in patients from Asia.

## Discussion

To some extent, molecular alterations such as genetic and epigenetic alterations in signaling pathways have changed the treatment of thyroid cancer through the development of targeted therapies. Many somatic genetic alterations including those in *BRAF, HRAS, KRAS, NRAS, PTEN*, and *HER1* have been revealed to play fundamental roles in the tumorigenesis of thyroid carcinoma. The close association of *TERT* promoter somatic mutations with tumorigenesis is widely recognized. *TERT* expression confers infinite proliferation potential and many other biological activities on cancer cells by maintaining telomere length^16^,^17^,^18^. In addition, recent data also demonstrated that the functional TERT rs2736100 SNP as a novel genetic component of PTC etiology^19^.

*TERT* promoter mutations, which might contribute to telomerase activation, have been reported in various cancers including melanoma, anaplastic oligodendroglioma, and bladder cancer^20^,^21^,^22^,^23^,^24^. Recently, the presence of *TERT* promoter somatic mutations has been reported in PTC; however, their diagnostic and prognostic significance remains unclear^7^,^8^. The present study aimed to determine the influence of *TERT* promoter somatic mutations on the clinicopathological outcomes and prognosis of PTC via a meta-analysis.

Larger tumor size, extrathyroidal invasion, stage III or IV disease, lymph node metastasis, and distant metastasis were correlated with poor prognostic features such as recurrence and short overall survival (OS)^15^,^25^. Several authors suggested that *TERT* promoter somatic mutations were associated with these aggressive clinicopathological characteristics^7^,^14^. This view is mainly in line with the results of our meta-analysis. Our findings illustrated that *TERT* promoter mutations were more likely to be present in patients with larger tumors, stage III or IV disease, lymph node metastasis, and distant metastasis. However, extrathyroidal invasion was at a critical level in terms of an association with *TERT* promoter mutations (P = 0.06).

One interesting finding in the current study was that the associations of *TERT* promoter mutations with multifocality and vascular invasion were not significant. This finding may be explained by the fact that the studies including data on focus number and vascular invasion were relatively small.

Most authors previously demonstrated that recurrence and poor OS were more likely to occur in patients with *TERT* promoter mutations than in those without mutations. By contrast, Qasem *et al*. found that the relationships of recurrence and short OS with *TERT* promoter mutations were not significant^14^. In our meta-analysis, however, patients with PTCs with *TERT* promoter mutations were more likely to experience recurrence and worse OS. Therefore, we conclude that *TERT* promoter mutations may represent an independent prognostic factor for prognosis in PTC.

A classic alteration found in PTC is the V600E mutation in the serine/threonine kinase *BRAF*^9^,^26^. Multicenter studies confirmed the association of *BRAF*^V600E^ with poor clinicopathological outcomes such as aggressive pathological features, increased recurrence, and resistance to radioiodine therapy in PTC^27^. Recently, a positive correlation between *TERT* promoter mutations and the *BRAF*^V600E^ mutation was reported in advanced PTCs, suggesting functional cooperation, and Xing *et al*. suggested that patients with PTC and coexistent *TERT* promoter and *BRAF*^V600E^ mutations tend to have poorer prognosis compared with patients harboring single mutations or wild-type *TERT* promoter and *BRAF*^7^. However, evidence reported by Biase *et al*. illustrated that *TERT* promoter mutations do not predict unfavorable features in papillary thyroid microcarcinoma, either alone or in combination with the *BRAF* V600E mutation^8^, and Melo *et al*. found no differences in persistence between tumors with *TERT* promoter mutations alone and those harboring both *TERT* and *BRAF* mutations^10^. Furthermore, Gandolfi *et al*. observed a positive association between *BRAF* V600E and *TERT* C228T mutations in a cohort of patients with PTC and distant metastases^11^. However, the associations of *BRAF* V600E with *TERT* C250T and all *TERT* promoter mutations were not significant. Our meta-analysis illustrated that *TERT* promoter mutations occurred more frequently in patients with *BRAF* mutations. Whether the coexistence of these two mutations predicts a poor prognosis is unclear and needs further investigation.

Our systematic review and meta-analysis assessed the associations of *TERT* promoter mutations with clinicopathological outcomes and prognosis in PTC. Although our study cannot identify a causal relationship between *TERT* promoter mutations and outcomes and *TERT* promoter mutations may be correlated with some other mutation or some confounder that may be even more useful, our findings may still be of great value in evidence-based clinical decision-making. In other words, our analysis should be interpreted with caution because of the heterogeneity of the data. Possible explanations for this heterogeneity include patient demographics as well as thyroidectomy, approaches to lymph node dissection, radioactive iodine (RAI) treatment, and the duration of follow-up. The population included patients from the USA, Portugal, Italy, and China, among other countries. Details of the approaches used for thyroidectomy, lymph node dissection, and RAI treatment were not all provided; furthermore, the outcomes might be interrelated. For instance, patients with more advanced disease tend to have extrathyroidal invasion, and thus, disease stage may confound the association between *TERT* promoter mutations and extrathyroidal invasion.

Apart from potential heterogeneity caused by varied management practices, differences in pathology reporting, follow-up duration, and the definition of remission may affect the conclusions of this meta-analysis. Another limitation is that the number of included articles is relatively small, and relevant unpublished data could not be obtained for further analysis. Therefore, our conclusions should be used cautiously. Larger studies would be helpful to definitely address the role of *TERT* promoter mutations in the prognosis of PTC. Furthermore, we did not distinguish between the C228T and C250T mutations from collective mutations in some studies^9^,^11^, and in the study by de Biase *et al*.^8^, only microcarcinomas were analyzed, which may have interfered with our results.

## Conclusion

Our meta-analysis demonstrated that *TERT* promoter mutations are closely associated with aggressive clinicopathological factors and poorer prognosis in PTC. *TERT* promoter mutations may be considered poor diagnostic and prognostic markers in PTC, and patients with such mutations may require aggressive management. More studies are needed to better evaluate the role of *TERT* promoter mutations in PTC.

## Materials and Methods

### Search strategy and literature selection

A systematical literature search was performed using online electronic databases (PubMed, EMBASE, and ISI Web of Science) for published papers through November 2015, and the search was supplemented by manual searching and reference backtracking using the following Medical Subject Headings and keywords: “TERT”, “ telomerase reverse transcriptase”, “mutation”, “thyroid”, “neoplasm(s)”, “tumor”, “cancer”, and “carcinoma”. The searches were limited to studies conducted in humans and written in English. Furthermore, the reference lists of retrieved articles were also reviewed to identify additional studies. Relevant unpublished data that were presented at international meetings such as the American Thyroid Association meeting were also included. We contacted the authors for additional tabular data when necessary^13^.

The following criteria were applied into the literature selection for studies that examined the association of *TERT* promoter mutations with high-risk clinic pathological factors and prognostic outcomes: (1) the studies had a randomized controlled trial or retrospective comparative study (cohort or case-control study) design; (2) the studies compared the *TERT* mutation-positive and *TERT* mutation-negative groups for patients with primary PTC; (3) the study investigated at least one outcome of interest; and (4) weighted mean differences (WMDs) and ORs with 95% CIs were reported or were available to be calculated. Studies lacking a control population, duplicates of previous publication, animal studies, abstracts, single-case reports, reviews, and unpublished reports were excluded. Additionally, studies that included *TERT* mutation analysis for patients with preoperative fine-needle aspiration biopsies were excluded to avoid false-negative results for the cytologic specimens. For studies with the same or overlapping data published by the same investigators, we selected studies with complete designs and larger sample sizes in our meta-analysis.

### Data extraction

All data were extracted independently by two authors (CP Liu and ZM Liu) and cross-checked to resolve any discrepancies. The following information was extracted from each included study regarding the association of *TERT* promoter mutations with clinical and pathological risk factors of PTC: first author, publication year, study location, ethnicity, number of cases, the percentage of females, age in both the *TERT* mutation-positive and *TERT* mutation-negative groups, *TERT* mutation rate, and the incidence rate of clinicopathological features in both the *TERT* mutation-positive and *TERT* mutation-negative groups. Regarding the associations of *TERT* promoter mutations with recurrent/persistent disease and mortality, the following data were extracted: first author, publication year, study location, number of follow-up cases, *TERT* mutation rate, stage of disease, initial treatment, poorer outcome, and the incidence rates of persistent/recurrence and mortality in both the *TERT* mutation-positive and *TERT* mutation-negative groups.

The following outcomes were extracted to compare the *TERT* mutation-positive and *TERT* mutation-negative groups for patients with primary PTC: the presence of extrathyroidal invasion, larger tumor size, advanced TNM stage (stage III or IV), unifocality, vascular invasion, lymph node metastasis, distant metastasis, *BRAF* mutation positivity, persistent/recurrent disease, and cancer-specific mortality. All of the procedures conformed to the guidelines for the meta-analysis of observational studies in epidemiology^28^.

### Statistical Analysis

The summary ORs with 95% CIs and WMDs with 95% CIs were calculated to compare dichotomous and continuous variables, respectively. The *χ*^2^-based Cochran’s Q statistic test and *I*^*2*^ statistics were used to evaluate heterogeneity between the studies, and heterogeneity was considered significant when *P* < 0.1 for the Q statistic or for an *I*^*2*^ statistic >50%^29^. A fixed-effects model (Mantel-Haenszel method) was used when no significant heterogeneity was detected; otherwise, a random-effects model (DerSimonian-Laird method) was applied. Subgroup analyses were also performed according to geographic region. In addition, we performed sensitivity analysis to assess the influence of each study on the overall estimate. Moreover, the potential publication bias was assessed using Egger’s test and funnel plot analysis. All meta-analyses were performed using Review Manager (version 5, Cochrane Collaboration, Oxford, UK). The *P*-values were two-tailed with a level of significant at 0.05.

## Additional Information

**How to cite this article**: Liu, C. *et al. TERT* promoter Mutation and Its Association with Clinicopathological Features and Prognosis of Papillary Thyroid Cancer: A Meta-analysis. *Sci. Rep.* **6**, 36990; doi: 10.1038/srep36990 (2016).

**Publisher’s note:** Springer Nature remains neutral with regard to jurisdictional claims in published maps and institutional affiliations.

## Figures and Tables

**Table 1:** Summary of the 10 Included Studies Comparing *TERT* Promoter Mutation Rates According to the Clinical and Pathological Risk Factors of PTC.

Study	Country	Ethnicity (A: Asians, C: Caucasians)	No. of patients with PTC	% Female	Age (*TERT* mutation-positive, *TERT* mutation-negative)	*TERT* Mutation Rate, %	*TERT* promoter mutation sequence	Clinicopathological features
Liu (T) 2013	Sweden	C	51	70.6	68 (49–84) 36 (22–97)	25.50%	N/A	LNM
Liu 2014	USA	A	408	78.2	53.40 ± 16.14, 43.66 ± 12.91	11.30%	N/A	Extrathyroidal invasion, tumor size, TNM stage, LNM, mortality
Gandolfi 2015	Italy	C	121	68.6	59.8 ± 17.1, 45.6 ± 17.6	17.40%	5′-GGATTCGCGGGCACAGAC-3′ 5′-AGCGCTGCCTGAAACTCG-3′	TNM stage, vascular invasion, LNM, DM, *BRAF* mutation, recurrence, mortality
Melo 2014	Portugal	C	332	77.7	58.4 ± 13.2, 43.6 ± 15.3	7.50%	N/A	Extrathyroidal invasion, tumor size, TNM stage, vascular, invasion, LNM, DM, *BRAF* mutation, mortality
Xing 2014	USA	C	507	72	51.7 ± 15.7, 45.1 ± 13.6	12.00%	N/A	Extrathyroidal invasion, TNM stage, unifocality, vascular invasion, LNM, DM, recurrence
Lee 2015	Korea	A	137	84.7	N/A	6.60%	5′-AGTGGATTC GCGGGCACAGA-3′ 5′-CAGCGCTGC CTG AAA CTC-3′	Extrathyroidal invasion, LNM,
Muzza 2015	Italy	C	182	77	57.6 (29–82), 44.2 (14–80)	12%	5′-AGTGGATTCGCGGGCACAGA-3′ 5′-GCAGCGCTGCCTGAAACTC-3′	Extrathyroidal invasion, TNM stage, unifocality, LNM, *BRAF* mutation, recurrence
Liu (XL) 2013	USA	C	257	N/A	N/A	11.70%	5′-AGTGGATTCGCGGGCACAGA-3′ 5′-CAGCGCTGCCTGAAACTC-3′	*BRAF* mutation
Qasem 2015	Arabia	C	138	75.8	37.4 ± 18.7, 30.8 ± 15.7	7.00%	5′-AGTGGATTCGCGGGCACAGA-3′ 5′-CAGCGCTGCCTGAAACTC-3′	Extrathyroidal invasion, tumor size, TNM stage, unifocality, vascular invasion, LNM, DM, recurrence
Biase 2015	Italy	C	404	78.2	50.2 ± 11.5, 47.8 ± 12.8	4.70%	5′-GGCTCCCAG TGG ATTCG-3′ 5′-AAGGAAGGG GAG GGG C-3′	TNM stage, unifocality, LNM, *BRAF* mutation, recurrence

N/A, not available; PTC, papillary thyroid cancer; LNM, lymph node metastasis; DM, distant metastasis.

**Table 2:** Summary of the Seven Included Studies Comparing the *TERT* promoter Mutation Rate in Patients with Poor Outcomes.

Study	Country	No. of Follow-up Cases	*TERT* promoter mutation Rate, %	Median Follow-up, months	Stage of Disease	Initial Treatment	Poor Outcome	Confirmation Method
Liu (T) 2013	Sweden	51	25.5%	N/A	III/IV 24.4	N/A	Mortality	N/A
Gandolfi 2015	Italy	121	17.4%	124.1	III/IV 43.0	TT, ipsilateral central neck dissection	Recurrence, persistent disease, and cancer-specific death	Pathology
Melo 2014	Portugal	284	7.5%	N/A	III/IV 64.7	N/A	Mortality	Radioiodine scan, Tg,
Xing 2014	USA	507	12.0%	24	III/IV 17.5	Therapeutic neck dissection and RAI ablation	Recurrence	Pathology, Radioiodine scan
Muzza 2015	Italy	182	12%	74.5	III/IV 20.6	N/A	Recurrence and persistence	Pathology, Radioiodine scan, Tg
Qasem 2015	Arabia	256	7.0%	34	III/IV 12.7	Near TT or TT, unilateral or bilateral neck dissection, RAI ablation	Recurrence	Pathology, Radioiodine scan, Tg
Biase 2015	Italy	288	4.7%	N/A	III/IV	N/A	Recurrence	N/A

N/A, not available; RAI, radioactive iodine; Tg, thyroglobulin; TT, total thyroidectomy; US, ultrasonography.

**Table 3:** Subgroup Analysis of the Effects of *TERT promoter mutations* on the Aggressive Clinicopathological Features and Poor Prognosis of PTC According to Country of Origin.

Subgroup	OR	95% CI	*I*^2^ (%)	Model used
Extrathyroidal invasion				
USA (one study)	4.92	2.76–8.78	0	—
Asia (three studies)	1.78	0.58–5.44	61	Random-effects
Europe (two studies)	1.21	0.61–2.40	8	Fixed-effects
Lymph node metastasis				
USA (one study)	2.87	1.65–4.98	0	—
Asia (three studies)	1.07	0.54–2.13	0	Fixed-effects
Europe (five studies)	2.01	0.98–4.13	53	Random-effects
Distant metastasis				
USA (one study)	10.68	4.39–25.99	0	—
Asia (one study)	0.54	0.03–9.93	0	—
Europe (two studies)	3.53	1.72–7.26	7	Fixed-effects
Recurrence				
USA (one study)	7.02	3.93–12.55	0	—
Asia (one study)	0.54	0.11–2.71	0	—
Europe (three studies)	4.76	2.43–9.30	0	Fixed-effects

OR, odds ratio; CI, confidence interval.
